# One-year safety and effectiveness of the Agent paclitaxel-coated balloon for the treatment of small vessel disease and in-stent restenosis

**DOI:** 10.1007/s12928-023-00953-8

**Published:** 2023-08-29

**Authors:** Masato Nakamura, Tsuyoshi Isawa, Shigeru Nakamura, Kenji Ando, Atsuo Namiki, Yoshisato Shibata, Toshiro Shinke, Yoshiaki Ito, Kenshi Fujii, Junya Shite, Ken Kozuma, Shigeru Saito, Junichi Yamaguchi, Seiji Yamazaki, Paul Underwood, Dominic J. Allocco

**Affiliations:** 1 Division of Minimally Invasive Treatment in Cardiovascular Medicine, Toho University Ohashi Medical Center, 2-22-36, Ohashi Meguro-ku, Tokyo, 153-8515 Japan; 2 Department of Cardiology, Sendai Kousei Hospital, Sendai, Miyagi Japan; 3 Cardiovascular Center, Kyoto-Katsura Hospital, Kyoto, Japan; 4 Department of Cardiology, Kokura Memorial Hospital, Fukuoka, Japan; 5 Department of Cardiology, Kanto Rosai Hospital, Kawasaki, Kanagawa Japan; 6 Department of Cardiology, Miyazaki Medical Association Hospital, Miyazaki, Japan; 7 Department of Cardiology, Showa University Hospital, Tokyo, Japan; 8 Department of Cardiology, Saiseikai Yokohama-City Eastern Hospital, Kawasaki, Kanagawa Japan; 9 Department of Cardiology, Sakurabashi Watanabe Hospital, Osaka, Japan; 10 Department of Cardiology, Osaka Saiseikai Nakatsu Hospital, Osaka, Japan; 11 Department of Cardiology, Teikyo University Hospital, Tokyo, Japan; 12 Heart Center, Shonan Kamakura General Hospital, Kawasaki, Kanagawa Japan; 13 Department of Cardiology, Tokyo Women’s Medical University Hospital, Tokyo, Japan; 14 Department of Cardiology, Sapporo Higashi Tokushukai Hospital, Sapporo, Hokkaido Japan; 15 Interventional Cardiology, Boston Scientific Corporation, Marlborough, MA USA

**Keywords:** Small vessel disease, In-stent restenosis, Drug-coated balloon

## Abstract

The Agent device consists of a semi-compliant balloon catheter, which is coated with a therapeutic low-dose formulation of paclitaxel (2 µg/mm^2^) blended with an inactive excipient acetyl-tri-*n*-butyl citrate (ATBC). AGENT Japan SV is a randomized controlled study that enrolled 150 patients from 14 Japanese sites treated with Agent or SeQuent Please paclitaxel-coated balloon. This study also includes a single-arm substudy evaluating the safety and effectiveness of Agent in patients with in-stent restenosis (ISR). Patients with a single de novo native lesion (lesion length ≤ 28 mm and reference diameter ≥ 2.00 to  < 3.00 mm) were randomized 2:1 to receive either Agent (*n* = 101) or SeQuent Please (*n* = 49). The ISR substudy enrolled 30 patients with lesion length ≤ 28 mm and reference diameter ≥ 2.00 to  ≤ 4.00 mm. In the SV RCT, target lesion failure (TLF) at 1 year occurred in four patients treated with Agent (4.0%) versus one patient with SeQuent Please (2.0%; *P* = 1.00). None of the patients in either treatment arm died. There were no significant differences in the rates of myocardial infarction, target lesion revascularization and target lesion thrombosis through 1 year. In the ISR substudy, the 1-year rates of TLF and target lesion thrombosis were 6.7% and 0.0%, respectively. These data support the safety and effectiveness of the Agent paclitaxel-coated balloon in patients with small vessels and ISR.

**Supplementary Information:**

The online version contains supplementary material available at 10.1007/s12928-023-00953-8.

## Introduction

Patients with coronary artery disease (CAD) have three common therapeutic options: (1) medical therapy and risk factor modification, (2) coronary artery bypass graft surgery (CABG) and (3) percutaneous coronary intervention (PCI). As PCI technology and revascularization procedures evolved, balloon angioplasty, bare metal stent (BMS) and drug-eluting stent (DES) succeeded each other as the primary catheter-based treatment for CAD. Recent evidence indicates that drug-coated balloons (DCBs) can also be safely used for the treatment of small vessel coronary lesions [1, 2]. The German [3] and Asia-Pacific [4] consensus groups recommend DCB treatment for ISR, de novo lesions in small coronary arteries and bifurcation lesions. Moreover, the International DCB Consensus Group [5] recently updated their guidelines to include DCB-only approach as a valid treatment alternative to DES for small vessel disease (SVD).

Drug delivery via DCB could result in a more homogeneous administration of the drug instead of creating a peri-strut gradient [6–10], reduced vascular smooth muscle cell proliferation [11] and a reduced rate of restenosis when compared to uncoated balloon treatment [12]. Drug concentration at the vessel wall with DCB is the highest at the time of injury when the neointimal process is the most vigorous. Afterward, the absence of drug and polymer (used in in-stent technologies) could help to facilitate re-endothelialization, thereby reducing the risk of late and very late thrombosis. Another advantage of DCB over DES is the decrease in duration of dual antiplatelet therapy (DAPT) when compared to DES, likely resulting in a reduced rate of bleeding-related complications [7, 10, 13]. Furthermore, it is an attractive approach in small vessels (SV), respecting the original anatomy of the arteries [9], and thus avoiding further reduction of the lumen diameter (as seen with stent struts [12]) and deployment of a permanent implant/prosthesis that could complicate future revascularization efforts [7, 10].

Agent uses the Emerge™ percutaneous transluminal coronary angioplasty (PTCA) balloon catheter platform (Boston Scientific Corporation) with a reduced paclitaxel dose density (2 μg/mm^2^) and an excipient that may reduce systemic drug exposure and associated vascular toxicity. The AGENT Japan SV study was designed to evaluate the safety and effectiveness of the Agent paclitaxel-coated balloon in SV de novo native lesions. Agent was non-inferior to SeQuent Please with regard to the primary end point of 6-month target lesion failure (TLF; 3.0% versus 0.0%, *P*_non-inferiority_ = 0.0012), with no significant differences in the rates of the individual components of TLF. AGENT Japan also includes a single-arm substudy evaluating the clinical safety and effectiveness of Agent in patients with ISR. This paper reports the 1-year follow-up data from the AGENT Japan SV RCT and ISR substudy.

## Methods

### Study design

AGENT Japan, a prospective, single-blind, non-inferiority study, randomized patients with SVD to receive either Agent or SeQuent Please in a 2:1 fashion. This trial also includes a single-arm substudy that evaluates the safety and effectiveness of Agent in patients with ISR of a previously treated lesion.

This study was approved by the Institutional Review Board at each site prior to enrollment and complied with the principles of the Declaration of Helsinki, Good Clinical Practice (GCP), Order for Enforcement of the Pharmaceutical and Medical Device Law and all applicable local and federal regulations. The AGENT Japan study is registered on clinicaltrials.gov under NCT04058990.

Study methods and primary end point data for the SV RCT and ISR substudy have been described previously [14]. Briefly, patients aged ≥ 20 years with target lesion located in a native coronary artery (SV RCT) or ISR of a previously treated lesion (ISR substudy) that was ≤ 28 mm in length were enrolled. Eligible patients had a reference vessel diameter (RVD) ≥ 2.00 mm to < 3.00 mm for the SV RCT and RVD ≥ 2.00 mm and ≤ 4.00 mm for the ISR substudy, as well as visually estimated target lesion stenosis ≥ 75 < 100%. Patients with left main disease, saphenous vein or arterial graft disease, complex bifurcation requiring treatment with more than one stent, severe calcification or thrombus in the target vessel were excluded. Patients who met the SV study selection criteria and underwent successful predilation of the target lesion were randomized 2:1 to Agent or SeQuent Please. Successful predilation was defined as < 30% visually estimated residual stenosis without major (NHLBI > Type D) flow-limiting dissection after the selected balloon was inflated at nominal pressure.

DAPT with aspirin and a P2Y12 inhibitor was prescribed for at least 3 months post-procedure. Clinical follow-up was scheduled at hospital discharge, 30 days, and 6 months after PCI, then annually between 1 and 5 years. Protocol-specified coronary angiography was required at baseline and at the 6-month follow-up.

### Study end points

The SV RCT primary end point was a non-inferiority comparison of Agent and SeQuent Please for the rate of TLF at 6 months (ischemia-driven revascularization of the target lesion [TLR], myocardial infarction [MI; Q-wave and non–Q-wave] related to the target vessel or cardiac death) [14]. For the ISR substudy, the primary end point of 1-year TLF was compared to a prespecified performance criterion. Additional prespecified clinical end points analyzed at 1 year included target vessel failure (TVF; composite of ischemia-driven TVR, MI related to the target vessel or cardiac death), all-cause death, MI (third universal definition) [15], target vessel revascularization (TVR), target lesion revascularization (TLR) and target lesion thrombosis (Academic Research Consortium definition) [15]. A clinical events committee reviewed and adjudicated all deaths, TVR, TLR, MI and target lesion thrombosis. Technical success rate was defined as the ability to cross and dilate the lesion to achieve residual angiographic stenosis no greater than 30% as confirmed by the angiographic core laboratory. Clinical procedural success was defined as technical success with no death or MI noted within 24 h of the index procedure.

Quantitative coronary analysis (QCA) was performed at baseline, post-procedure and at the 6-month follow-up visit by an independent core laboratory (Beth Israel Deaconess Medical Center, Boston, MA, USA). Six-month angiographic end points included minimum lumen diameter, late lumen loss, % diameter stenosis and binary restenosis. Late lumen loss was calculated as the post-procedure MLD minus 6-month follow-up MLD. Late lumen enlargement (LLE) was defined as late lumen loss less than zero. Binary restenosis was a diameter stenosis of > 50%. Functional status of general health-related quality of life was measured by changes in EQ-5D scores at hospital discharge, 6 months and 1, 2 and 3 years after the index procedure.

### Statistical analysis

Discrete variables were reported as percentages (%), and differences were assessed using Chi-squared or Fisher’s exact tests. Continuous variables were calculated as the mean ± SD and compared using Student’s *t* test. Statistical analyses were conducted using SAS^®^ Version 9.2 (SAS Institute, Cary, NC, USA).

## Results

### AGENT Japan SV randomized control trial

**Fig. 1:**
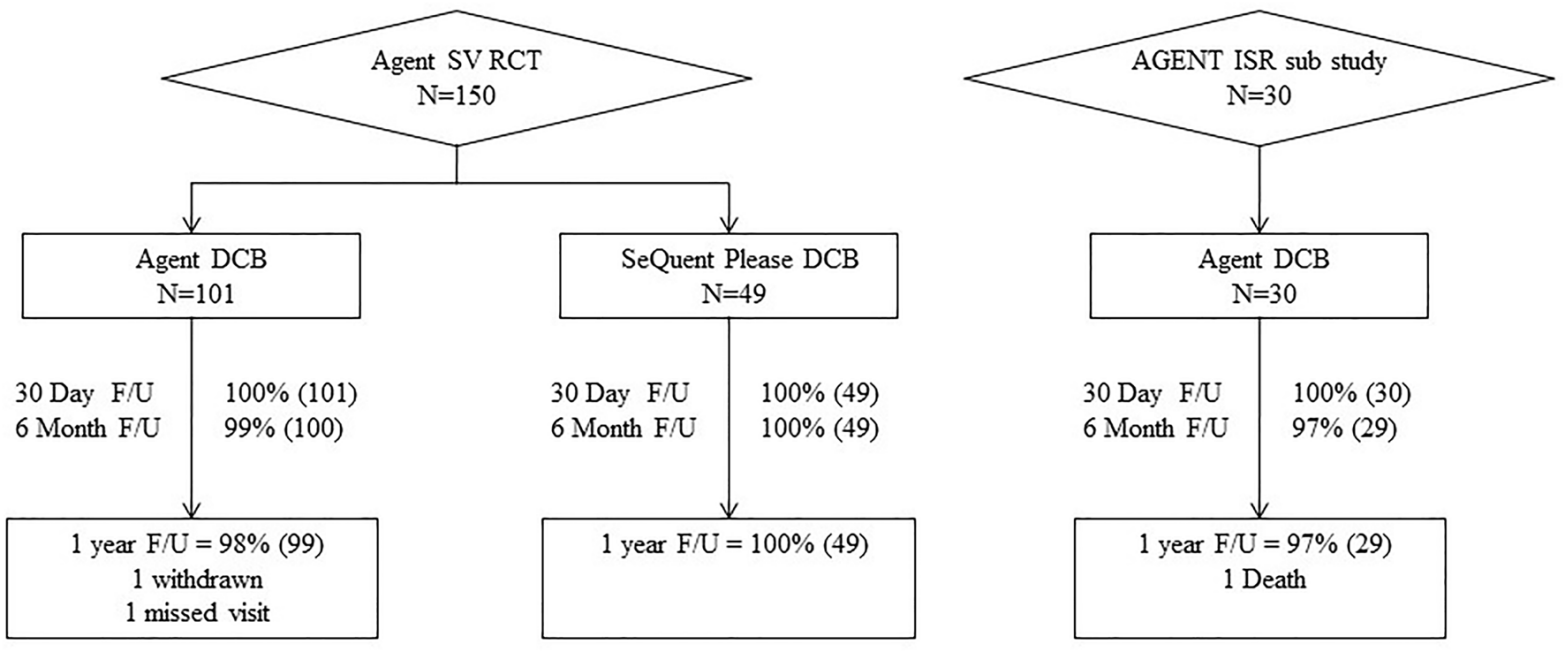
AGENT Japan enrollment and follow-up. *F/U* follow-up; *ISR* in-stent restenosis, *RCT* randomized control trial, *SV* small vessel

A total of 150 patients were enrolled and randomized at 14 sites in Japan. Of these, 101 were randomized to Agent and 49 to SeQuent Please (Fig. 1). One-year follow-up was available in 99 (98.0%) Agent- and 49 (100%) SeQuent Please-treated patients (Fig. 1). Baseline patient clinical demographics and quantitative coronary angiographic characteristics were similar between treatment groups (Table 1). The average age was 68 years, 24% were female and 34% patients had medically treated diabetes mellitus (Table 1). Seventy percent of patients in the Agent arm and 67% in the SeQuent Please arm were classified as American Heart Association/American College of Cardiology Type B2 or C lesions (Table 1) by the angiographic core laboratory. Procedural characteristics (Table 2) and post-procedural angiographic results (Table 2) were similar between treatment groups, as well as the rates of technical and clinical success (Table 2). As indicated in Table 3, DAPT (aspirin plus a P2Y12 inhibitor) usage at 1 year was 18% in the Agent and 31% in the SeQuent Please arms, respectively (*P* = 0.08).

**Table 1:** Baseline characteristics

	Small vessel study		ISR Substudy
	Agent *N* = 101 patients	SeQuent Please *N* = 49 patients	Agent *N* = 30 patients
Patient characteristics			
Male	74.3% (75)	77.6% (38)	86.7% (26)
Current smoker	21.8% (22)	10.2% (5)	20.0% (6)
Diabetes	35.6% (36)	34.7% (17)	43.3% (13)
Hyperlipidemia	88.1% (89)	95.9% (47)	96.7% (29)
Hypertension	76.2% (77)	73.5% (36)	80.0% (24)
Myocardial infarction	30.7% (31)	28.6% (14)	56.7% (17)
Angina status			
Stable	97.0% (98)	91.8% (45)	96.7% (29)
Unstable	3.0% (3)	8.2% (4)	3.3% (1)
Target vessel treated^a^	*N* = 101 lesions	*N* = 49 lesions	*N* = 30 lesions
LAD	27.7% (28)	22.4% (11)	60.0% (18)
LCx	42.6% (43)	51.0% (25)	16.7% (5)
RCA	29.7% (30)	26.5% (13)	23.3% (7)
Target lesion characteristics^a^			
RVD, mm	2.19 ± 0.37 (101)	2.22 ± 0.34 (49)	2.63 ± 0.59 (30)
MLD, mm	0.70 ± 0.28 (101)	0.71 ± 0.27 (49)	0.96 ± 0.45 (30)
Diameter stenosis, %	68.23 ± 10.95 (101)	68.02 ± 10.52 (49)	64.15 ± 12.01 (30)
Lesion length, mm	12.58 ± 5.94 (100)	13.38 ± 5.70 (49)	15.27 ± 6.41 (30)
			11.03 ± 4.68 (30)^b^
Modified ACC/AHA B2/C	70.3% (71)	67.3% (33)	76.7% (23)

Intent-to-treat analysis; values are in % or mean ± standard deviation

*ACC/AHA* American College of Cardiology/American Heart Association, *LAD* left anterior descending, *LCx* left circumflex, *MLD* minimum lumen diameter, *RCA* right coronary artery, *RVD* reference vessel diameter

^a^As determined by the angiographic core laboratory

^b^ISR lengths (ISR substudy); *P* values not significant

**Table 2:** Procedural and angiographic outcomes

	Small vessel study		ISR substudy
	Agent *N* = 101 patients *N* = 101 lesions	SeQuent Please *N* = 49 patients *N* = 49 lesions	Agent *N* = 30 patients *N* = 30 lesions
Procedural characteristics			
Technical success^A^	66.3% (67)	77.6% (38)	93.3% (28)
Clinical procedural success^B^	66.3% (67)	77.6% (38)	93.3% (28)
Total DCB length deployed^C^ (mm)	19.28 ± 5.85 (101)	18.61 ± 4.36 (49)	22.13 ± 6.54 (30)
Pre-dilatation^C^, %	100% (101)	100% (49)	100% (30)
Post-dilatation^C^, %	1.0% (1)	0.0% (0)	0.0% (0)
Maximum pressure overall^C^ (atm)	11.40 ± 3.06 (101)	12.65 ± 3.63 (49)	14.77 ± 4.90 (30)
Post-procedural characteristics			
MLD, mm			
In-lesion/in-stent^D^	1.69 ± 0.33 (101)	1.67 ± 0.30 (49)	2.10 ± 0.46 (30)
In-segment	1.72 ± 0.35 (101)	1.71 ± 0.32 (49)	2.17 ± 0.47 (30)
DS, %DS, %			
In-lesion/in-stent^D^	23.04 ± 10.51 (101)	23.63 ± 13.23 (49)	20.26 ± 6.88 (30)
In-segment	21.77 ± 11.02 (101)	21.83 ± 14.37 (49)	17.39 ± 7.66 (30)
Acute gain			
In-lesion/in-stent^D^	0.99 ± 0.37 (101)	0.97 ± 0.31 (49)	1.14 ± 0.33 (30)
In-segment	1.02 ± 0.38 (101)	1.00 ± 0.34 (49)	1.22 ± 0.37 (30)
6 months			
RVD, mm	2.22 ± 0.36 (99)	2.20 ± 0.36 (48)	2.59 ± 0.50 (29)
MLD, mm			
In-lesion/in-stent^D^	1.72 ± 0.38 (99)	1.65 ± 0.41 (48)	1.99 ± 0.49 (29)
In-segment	1.75 ± 0.40 (99)	1.70 ± 0.41 (48)	2.04 ± 0.50 (29)
DS, %			
In-lesion/in-stent^D^	22.34 ± 12.91 (99)	24.52 ± 16.03 (48)	23.34 ± 10.27 (29)
In-segment	20.58 ± 14.01 (99)	22.25 ± 16.47 (48)	21.46 ± 10.98 (29)
Late loss			
In-lesion/in-stent^D^	-0.03 ± 0.34 (99)	0.03 ± 0.34 (48)	0.07 ± 0.29 (29)
In-segment	-0.04 ± 0.36 (99)	0.02 ± 0.35 (48)	0.10 ± 0.32 (29)
LLE			
In-lesion/in-stent^D^	58.6% (58)	47.9% (23)	34.5% (10)
In-segment	56.6% (56)	43.8% (21)	41.4% (12)
Binary restenosis			
In-lesion/in-stent^D^	5.1% (5)	10.4% (5)	3.4% (1)
In-segment	5.1% (5)	8.3% (4)	3.4% (1)

Intent-to-treat analysis; values are mean ± standard deviation

*DS* diameter stenosis, *MLD* minimum lumen diameter, *RVD* reference vessel diameter

^A^Residual angiographic stenosis no greater than 30% as determined by the core laboratory

^B^Technical success with no death/MI within 24 h of yhe index procedure

^C^By target lesion

^D^In-stent analysis (ISR substudy); *P* values not significant

**Table 3:** Antiplatelet medications through 1 year

	Small vessel study			ISR substudy
	Agent *N* = 101 patients	SeQuent Please *N* = 49 patients	*P* value	Agent *N* = 30 patients
Aspirin				
Discharge	98.0% (99)	100.0% (49)	1.00*	100.0% (30)
3 months	94.1% (95)	95.9% (47)	1.00*	96.6% (28)
6 months	74.3% (75)	87.8% (43)	0.06	89.7% (26)
1 year	57.0% (57)	67.3% (33)	0.23	72.4% (21)
Dual antiplatelet therapy				
Discharge	95.0% (96)	95.9% (47)	1.00*	100.0% (30)
3 months	87.1% (88)	91.8% (45)	0.39	96.6% (28)
6 months	55.4% (56)	73.5% (36)	0.03	72.4% (21)
1 year	18.0% (18)	30.6% (15)	0.08	48.3% (14)

Intent-to-treat subjects; values are in %

*P values are two-sided from Fisher's exact test; *P* values without an asterisk are from the Chi-square test; dual antiplatelet therapy (DAPT) = aspirin and one of clopidogrel, ticlopidine, prasugrel or ticagrelor

### Angiographic follow-up

Angiographic follow-up was performed after 6 months for all patients except 2 in the Agent arm and 1 in the SeQuent Please arm (follow-up rates 98% and 98% in Agent and SeQuent Please arms, respectively). Baseline, post-procedural lesions, and 6 months angiographic follow-up, quantified by coronary angiography, revealed no significant differences between the two treatment arms (Table 1). At 6 months, negative late loss was observed in the Agent arm (Agent − 0.03 ± 0.34 mm vs. SeQuent Please 0.03 ± 0.34 mm; *P* = 0.31; Table 2), and in-lesion LLE occurred in 59% of patients in the Agent versus 48% of SeQuent Please arm (*P* = 0.22; Table 2).

### Clinical events

**Fig. 2:**
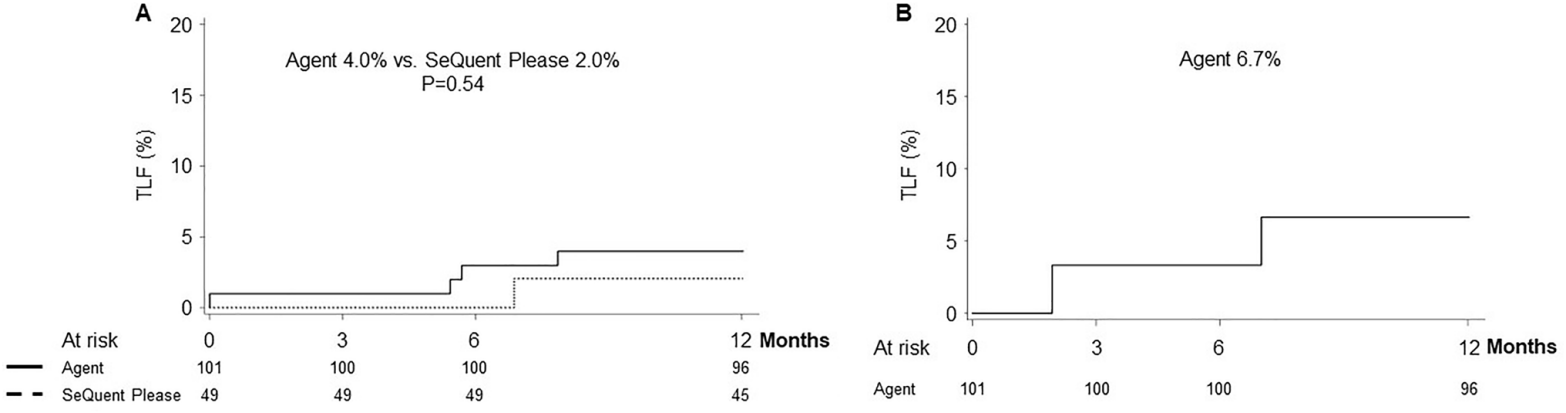
Target lesion failure (TLF) through 1 year of follow-up. Time-to-event curves for AGENT Japan **A** SV RCT and **B** ISR substudy. *ISR* in-stent restenosis, *SV* small vessel. *P* value from log-rank test for the SV RCT

As reported previously, the primary end point of 6-month TLF was met: Agent was non-inferior to SeQuent Please DCB (3.0% vs. 0.0%, respectively; *P* = 0.0012 for non-inferiority) [14]. TLF rates over the 1-year period were similar between the 2 DCBs (Agent 4.0% vs. SeQuent Please 2.0%; *P* = 1.00; Fig. 2). One patient in the Agent arm had a peri-procedural MI related to the target vessel and underwent TLR; two patients in Agent and 1 patient in the SeQuent Please arms underwent TLR that was treated with PCI (165, 173 and 209 days post-procedure, respectively). Additionally, another patient in the Agent arm had a TLR 228 days post-procedure that was treated with PCI. This patient also experienced a sub-acute (2–30 days) target lesion thrombosis, a non-Q-wave-MI related to the target vessel and underwent TLR 239 days post-procedure that was treated with PCI. No target lesion thrombosis occurred in the SeQuent Please arm (Agent 1.0% vs. SeQuent Please 0.0%; *P* = 1.00). There were no deaths in either treatment arm. Additional clinical end points shown in Table 4 were comparable between study arms.

**Table 4:** Clinical outcomes through 1 year

	Small vessel study		ISR substudy
	Agent *N* = 99 patients	SeQuent Please *N* = 49 patients	Agent *N* = 30 patients
All death/MI	2.0% (2)	0.0% (0)	3.3% (1)
Death	0.0% (0)	0.0% (0)	3.3% (1)
Cardiac death	0.0% (0)	0.0% (0)	3.3% (1)
Non-cardiac death	0.0% (0)	0.0% (0)	0.0% (0)
MI	2.0% (2)	0.0% (0)	3.3% (1)
Q-wave	0.0% (0)	0.0% (0)	0.0% (0)
Non-Q-wave	2.0% (2)	0.0% (0)	3.3% (1)
Related to TV	2.0% (2)	0.0% (0)	3.3% (1)
TVR	6.1% (6)	2.0% (1)	6.7% (2)
TLR	4.0% (4)	2.0% (1)	3.3% (1)
TLF	4.0% (4)	2.0% (1)	6.7% (2)
TVF	6.1% (6)	2.0% (1)	10.0% (3)
Definite or probable thrombosis related to target lesion	1.0% (1)	0.0% (0)	0.0% (0)
Definite ST	1.0% (1)	0.0% (0)	0.0% (0)
Probable ST	0.0% (0)	0.0% (0)	0.0% (0)

Intent-to-treat subjects; values are in % (*n*); time-to-event analysis; *P* values were not significant. TVF was defined as composite of any ischemia-driven TVR, MI related to the target vessel or cardiac death

*MI* myocardial infarction, *ST* stent thrombosis, *TLF* target lesion failure, *TLR* target lesion revascularization, *TV* target vessel, *TVF* target vessel failure, *TVR* target vessel revascularization

Agent and SeQuent Please DCBs were associated with similar improvements in quality of life, as assessed using the EQ-5D questionnaires (Supplementary Table 1). The EQ-5D scores improved post-procedure and this improvement was sustained through 1 year of follow-up.

### AGENT Japan ISR substudy

A total of 30 patients were enrolled in the ISR substudy at nine sites in Japan. One-year follow-up was available in 29 (97%) patients treated with the Agent DCB (Fig. 1). Baseline patient clinical demographics and quantitative coronary angiographic characteristics are shown in Table 1. Briefly, the mean age of subjects was 69 years, 13% were female and 43% had diabetes. Seventy-seven percent of patients were classified as AHA/ACC Type B2 or C lesions (Table 1). Procedural characteristics, post-procedural angiographic results and the rates of technical and clinical success are shown in Table 2. As indicated in Table 3, DAPT usage at 1 year was 48%.

### Angiographic follow-up

Angiographic follow-up was performed at 6 months in all but one patient (follow-up rate 97%). Quantitative coronary angiographic lesion characteristics and 6 months angiographic follow-up are shown in Table 2. At 6 months, in-lesion LLE occurred in 10 of 29 patients treated with Agent (Table 2).

### Clinical events

As reported previously, the primary end point of 6-month TLF was observed in 3.3% of Agent-treated patients, which was significantly less than the study success criterion of 15.1% (1-sided 97.5% UCB: 9.8%, *P* < 0.0001) [14]. In the ISR cohort at 1 year, TLF occurred in two patients (6.7%). One patient had a non-Q-wave MI related to the target vessel 59 days post-procedure and died suddenly that was adjudicated as cardiac related (not related to ST). Additionally, another patient had a TLR 213 days post-procedure that was treated with PCI. None of the patients experienced target lesion thrombosis through 1 year of follow-up (Table 4). There were general improvements in the quality-of-life scores from baseline to index procedure, which were sustained through 1 year (Supplementary Table 2).

## Discussion

The AGENT Japan study represents the first clinical trial comparing Agent and SeQuent Please DCBs with two different drug formulations in Japanese patients. The principal findings through 1 year include: (1) relatively comparable TLF rates between arms; (2) no significant differences in the rates of individual components of TLF (cardiac death, TV-MI and TLR); (3) rare occurrence of target lesion thrombosis (1.0% Agent vs. 0.0% SeQuent Please); (4) low event rates in patients with ISR with no incidence of device-related target lesion thrombosis through 1 year of follow-up.

PCI is the most common treatment for patients with symptomatic CAD. The optimal management of patients presenting with de novo SVD is clinically challenging due to vessel size and difficulties with device delivery. In addition, the issue of ISR following implantation of a BMS or DES remains a clinical challenge, due to late stent thrombosis, dependency on prolonged dual antiplatelet therapy and continued restenosis, leading to a quest for an alternative therapy. DCB technology was developed as an alternative to stent-based treatment for the management of atherosclerotic CAD and ISR. Currently available non-stent therapeutic drug delivery options include angioplasty balloons coated with the antiproliferative drug paclitaxel. DCBs may present an advantage over DES in that they do not introduce an additional stent layer, thereby potentially reducing neointimal proliferation, lumen impingement and mechanical complications (e.g., fracture, malposition, thrombosis) [16], which may account for the favorable outcomes observed in these SVD and ISR populations previously treated with BMS or DES who are known to be sub-optimal candidates for stent placement.

The Agent DCB is coated with a formulation of paclitaxel and a highly efficient ATBC [17] excipient in a drug dose density of 2 μg/mm^2^. This paclitaxel coating, once delivered to the arterial tissue, resists the initiation of restenosis by reducing the inflammation caused during the widening of the stenosis. The Agent device incorporates a number of features designed to allow a reduced paclitaxel dose density (2 µm/mm^2^) as compared to the majority of currently marketed products, such as SeQuent Please DCB (3 µm/mm^2^) (B. Braun Interventional Systems Inc.).

Data from the AGENT Japan SV RCT are consistent with those reported previously. In the PICCOLETO II trial [18], paclitaxel-eluting balloons versus DES showed lower rates of MACE (5.6% vs. 7.5% of patients; *P* = 0.55) and vessel thrombosis (0.0% vs. 1.9%; *P* = 0.15) in patients with SVD. #Results from the BASKET-SMALL 2 [19] study (The Basel Kosten Effektivitäts Trial) demonstrated non-inferiority of paclitaxel-eluting DCB versus DES with regard to the primary end point of 1-year MACE (8.0% in both groups) in patients with small vessel CAD (*P* = 0.0217). One-year TVR occurred in 3.4% versus 4.5% patients (*P* = 0.4375), with no significant differences in the rates of definite/probable stent thrombosis. The BELLO [20] (Balloon Elution and Late Loss Optimization) study evaluated the efficacy of paclitaxel-eluting DCB compared with DES for the reduction of restenosis in small vessels. The 1-year TLR [21] rate was 6.7% in the DCB group compared to 12.1% in the DES group (*P* = 0.23). The rate of 1-year TLR in patients with SVD who were treated with paclitaxel-coated balloon versus zotarolimus-eluting stents during the RESTORE SVD (The Basel Kosten Effektivitäts Trial) trial was 4.4% versus 2.6%, respectively (*P* = 0.72) [22].

The rate of 1-year TLR in the Agent Japan ISR substudy was 3.3%, which is considerably lower than that reported in the AGENT ISR (27.7%) and ISAR (22.1%; Intracoronary Stenting and Angiographic Results: Drug-Eluting Stent In-Stent Restenosis, 3A study [23]) studies. The exact reasons explaining the low TLR of ISRs in this study are difficult to determine, since the small sample size may have limited the ability to measure infrequent clinical adverse outcomes. Therefore, further large-scale studies are needed to confirm the findings of this study. Agent IDE, a multicenter randomized controlled trial, will test the superiority of Agent DCB versus plain old balloon angioplasty for the treatment of ISR [17]. The study has finished enrollment and 1-year follow-up for the primary end point is ongoing.

The QOL response to treatment appeared similar in both Agent and SeQuent Please arms. The initial improvement seen at 6 months plateaued by 1 year; however, pain/discomfort and anxiety/depression scores remained favorable compared to baseline. These are in line with the favorable long-term clinical outcomes demonstrated in both groups. This finding is consistent with that PCI leads to a more rapid recovery and improved short-term health status compared to CABG [24]. The response in the ISR substudy was similar, but not as robust. This may be a reflection of the more advanced coronary artery disease experienced by the ISR patients and the sense that recurrent stenosis may jeopardize their future.

DAPT with aspirin and a P2Y12 inhibitor was prescribed for at least 3 months post-procedure, after which these medications were continued at the treating physicians’ discretion. At 1 year, 18% in the Agent and 31% in the SeQuent Please arms were taking DAPT. In the ISR substudy, 48% of patients were taking the DAPT at 1 year. DAPT duration is often influenced by clinical factors observed by the treating physician. This finding seems consistent with a substudy of the BASKET-SMALL 2 trial, which found that the use of DCB had shorter DAPT and less major bleeding compared to DES [25]. Additionally, the SeQuent Please post-marketing surveillance study for ISR lesions had similarly high DAPT usage 93% at 3 months and 80% at 1 year. Thus, the clinical scenario may have influenced the physician to prolong the administration of DAPT. Furthermore, this may suggest that stent-less strategies are more likely to shorten DAPT. Another possibility is that the attending physician was trying to avoid ischemic events due to early interruption of DAPT because of the nature of the trial. Indeed, the optimal duration of DAPT in DCB has not been established.

### Study limitations

45 treated cases counted toward technical failure based on the definition of angiographic success. Of these, two patients in the Agent arm underwent TVR (195 days after the index procedure) and TLR (228 days after the index procedure). None of the 11 patients in the SeQuent Please group experienced events related to the target vessel. This finding suggests that the definition of optimal dilatation after DCB may require further investigation.

The previously reported SV RCT primary end point was a non-inferiority comparison of Agent and SeQuent Please for the rate of TLF at 6 months [14]. This study is not designed to assess non-inferiority at 1 year. Although the AGENT Japan SV study is an RCT, a relatively small number of patients were enrolled. Patients with high complexity were excluded from this study based on the protocol’s inclusion/exclusion criteria, and therefore this analysis does not fully represent real-world clinical practice. There is potential for bias due to differences in treatment patterns or patient complexity. Results obtained may not apply to patient and lesion types excluded from enrollment. Finally, the ISR substudy includes a non-randomized patient population and uses a prespecified study success criterion; this substudy is not adequately powered to draw definitive conclusions.

## Conclusions

AGENT Japan is the first randomized controlled trial that compares the Agent balloon coated with a low-dose formulation of paclitaxel (2 μg/mm^2^) with the SeQuent Please paclitaxel-coated balloon (3 μg/mm^2^) for the treatment of SVD. The 1-year outcomes of Agent SV RCT demonstrate favorable safety and efficacy of Agent DCB in Japanese patients with SVD. Clinical event rates were low and comparable between Agent and SeQuent Please treatment arms. Also, event rates in the ISR substudy remained low through 1 year, with no incidence of device-related target lesion thrombosis. These data support the use of Agent paclitaxel-eluting balloon for the treatment of both SVD and ISR.

### Supplementary Information

Below is the link to the electronic supplementary material.

Supplementary file1 (DOCX 48 kb)

## Data Availability

The deidentified participant data for this clinical trial will not be shared.

## References

[1] Tian J, da Tang Y, Qiao S (2020). Two-year follow-up of a randomized multicenter study comparing a drug-coated balloon with a drug-eluting stent in native small coronary vessels: the RESTORE Small Vessel Disease China trial. Catheter Cardiovasc Interv.

[2] Jeger RV, Farah A, Ohlow MA (2018). Drug-coated balloons for small coronary artery disease (BASKET-SMALL 2): an open-label randomised non-inferiority trial. Lancet.

[3] Kleber FX, Mathey DG, Rittger H, Scheller B, German Drug-eluting Balloon Consensus Group (2011). How to use the drug-eluting balloon: recommendations by the German consensus group. EuroIntervention.

[4] Her A-Y, Shin E-S, Bang LH (2021). Drug-coated balloon treatment in coronary artery disease: Recommendations from an Asia-Pacific Consensus Group. Cardiol J.

[5] Jeger RV, Eccleshall S, Wan Ahmad WA (2020). Drug-coated balloons for coronary artery disease: third report of the International DCB Consensus Group. JACC Cardiovasc Interv.

[6] De Labriolle A, Pakala R, Bonello L, Lemesle G, Scheinowitz M, Waksman R (2009). Paclitaxel-eluting balloon: from bench to bed. Catheter Cardiovasc Interv.

[7] Cortese B, Bertoletti A (2012). Paclitaxel coated balloons for coronary artery interventions: a comprehensive review of preclinical and clinical data. Int J Cardiol.

[8] Gutiérrez-Chico JL, van Geuns RJ, Koch KT, Koolen JJ, Duckers H, Regar E, Serruys PW (2011). Paclitaxel-coated balloon in combination with bare metal stent for treatment of de novo coronary lesions: an optical coherence tomography first-in-human randomised trial, balloon first vs. stent first. EuroIntervention.

[9] Belkacemi A, Agostoni P, Voskuil M, Stella PR (2011). Coronary bifurcation lesions treated with the drug-eluting balloon: a preliminary insight from the DEBIUT study. EuroIntervention.

[10] Ahmed W, Shah MA, Thaver AM, Mirza J (2011). Drug-eluting balloon (DEB) for de-novo coronary artery disease and in-stent restenosis: immediate and intermediate term results from a prospective registry. J Pak Med Assoc.

[11] Ali RM, Degenhardt R, Zambahari R, Tresukosol D, Ahmad WAW, Kamar HBH, Kui-Hian S, Ong TK, bin Ismail O, bin Elis S, Udychalerm W, Ackermann H, Boxberger M, Unverdorben M (2011). Paclitaxel-eluting balloon angioplasty and cobalt chromium stents versus conventional angioplasty and paclitaxel-eluting stents in the treatment of native coronary artery stenoses in patients with diabetes mellitus. EuroIntervention.

[12] Loh JP, Waksman R (2012). Paclitaxel drug-coated balloons: a review of current status and emerging applications in native coronary artery de novo lesions. JACC Cardiovasc Interv.

[13] Bonaventura K, Sonntag S, Kleber FX (2011). Antiplatelet therapy in the era of percutaneous coronary intervention with drug-eluting balloons. EuroIntervention.

[14] Nakamura M (2023). Drug-coated balloon for the treatment of small vessel coronary artery disease—a randomized non-inferiority trial. Circ J.

[15] Cutlip DE, Windecker S, Mehran R, Boam A, Cohen DJ, van Es GA (2007). Clinical end points in coronary stent trials: a case for standardized definitions. Circulation.

[16] Siontis GCM, Stefanini GG, Mavridis D (2015). Percutaneous coronary interventional strategies for treatment of in-stent restenosis: a network meta-analysis. Lancet.

[17] Yeh RW, Bachinsky W, Stoler R, Bateman C, Tremmel JA, Abbott JD (2021). Rationale and design of a randomized study comparing the agent drug coated balloon to plain old balloon angioplasty in patients with in-stent restenosis. Am Heart J.

[18] Cortese B, Di Palma G, Guimaraes MG, Piraino D, Orrego PS, Buccheri D (2020). Drug-coated balloon versus drug-eluting stent for small coronary vessel disease: PICCOLETO II randomized clinical trial. JACC Cardiovasc Interv.

[19] Jeger RV, Farah A, Ohlow MA, Mangner N, Möbius-Winkler S, Leibundgut G (2018). Drug-coated balloons for small coronary artery disease (BASKET-SMALL 2): an open-label randomized non-inferiority trial. Lancet.

[20] Latib A, Colombo A, Castriota F, Micari A, Cremonesi A, De Felice F (2012). A randomized multicenter study comparing a paclitaxel drug-eluting balloon with a paclitaxel-eluting stent in small coronary vessels: the BELLO (Balloon Elution and Late Loss Optimization) study. J Am Coll Cardiol.

[21] Naganuma T, Latib A, Sgueglia GA (2015). A 2-year follow-up of a randomized multicenter study comparing a paclitaxel drug-eluting balloon with a paclitaxel-eluting stent in small coronary vessels the BELLO study. Int J Cardiol.

[22] Tang Y, Qiao S, Su X, Chen Y, Jin Z, Chen H (2018). Drug-coated balloon versus drug-eluting stent for small-vessel disease: the RESTORE SVD China randomized trial. JACC Cardiovasc Interv.

[23] Sebastian K, Himanshu R, Jens W, Felix A, Stylianos P, Michael J (2022). A prospective trial of a novel low-dose paclitaxel-coated balloon therapy in patients with restenosis in drug-eluting coronary stents intracoronary stenting and angiographic results: optimizing treatment of drug eluting stent in-stent REstenosis 3A (ISAR-DESIRE 3A). Catheter Cardiovasc Interv.

[24] Alexander K (2017). Quality of life after coronary artery bypass graft surgery versus percutaneous coronary intervention what do the trials tell us?. Curr Opin Cardiol.

[25] Felix M, Ahmed F, Marc-Alexander O, Norman M, Jochen W (2022). Drug-coated balloons for small coronary artery disease in patients with chronic kidney disease: a pre-specifed analysis of the BASKET-SMALL 2 trial. Clin Res Cardiol.

