# Supplementary material for: One-year safety and effectiveness of the Agent paclitaxel-coated balloon for the treatment of small vessel disease and in-stent restenosis
Source: Cardiovasc Interv Ther. 2023 Aug 29;39(1):47–56. doi: 10.1007/s12928-023-00953-8 (PMC10764532; doi:10.1007/s12928-023-00953-8)
Supplement: Supplementary file 1 — Supplementary file1 (DOCX 48 kb) [file 12928_2023_953_MOESM1_ESM.docx]

# Supplementary Tables

**Supplementary Table 1. Quality of Life (SV Study)**

| **EQ-5D** | **Agent DCB** | | | | |  | **SeQuent Please DCB** | | | | |
| --- | --- | --- | --- | --- | --- | --- | --- | --- | --- | --- | --- |
|  | **Baseline**  **N=101 patients** | **6 months N=100 patients** | | **1 year N=99 patients** | |  | **Baseline**  **N=49 patients** | **6 months N=49 patients** | | **1 year N=49 patients** | |
| **Mobility** |  |  | |  | |  |  |  | |  | |
| No problems | 91.1% (92) | 94.0% (94) | | 90.9% (90) | |  | 91.8% (45) | 95.9% (47) | | 87.8% (43) | |
| Slight problems | 5.9% (6) | 5.0% (5) | | 6.1% (6) | |  | 8.2% (4) | 2.0% (1) | | 12.2% (6) | |
| Moderate problems | 3.0% (3) | 1.0% (1) | | 2.0% (2) | |  | 0.0% (0) | 0.0% (0) | | 0.0% (0) | |
| Severe problems | 0.0% (0) | 0.0% (0) | | 0.0% (0) | |  | 0.0% (0) | 2.0% (1) | | 0.0% (0) | |
| Unable | 0.0% (0) | 0.0% (0) | | 1.0% (1) | |  | 0.0% (0) | 0.0% (0) | | 0.0% (0) | |
| **Self-Care** |  | |  | |  |  |  | |  | | |
| No problems | 98.0% (99) | 97.0% (97) | | 96.0% (95) | |  | 100% (49) | 98.0% (48) | | 98.0% (48) | |
| Slight problems | 1.0% (1) | 3.0% (3) | | 3.0% (3) | |  | 0.0% (0) | 2.0% (1) | | 2.0% (1) | |
| Moderate problems | 1.0% (1) | 0.0% (0) | | 0.0% (0) | |  | 0.0% (0) | 0.0% (0) | | 0.0% (0) | |
| Severe problems | 0.0% (0) | 0.0% (0) | | 0.0% (0) | |  | 0.0% (0) | 0.0% (0) | | 0.0% (0) | |
| Unable | 0.0% (0) | 0.0% (0) | | 1.0% (1) | |  | 0.0% (0) | 0.0% (0) | | 0.0% (0) | |
| **Usual Activities** |  |  | |  | |  |  | |  | |  |
| No problems | 94.1% (95) | 97.0% (97) | | 97.0% (96) | |  | 93.9% (46) | 95.9% (47) | | 91.8% (45) | |
| Slight problems | 4.0% (4) | 1.0% (1) | | 1.0% (1) | |  | 6.1% (3) | 4.1% (2) | | 8.2% (4) | |
| Moderate problems | 2.0% (2) | 2.0% (2) | | 1.0% (1) | |  | 0.0% (0) | 0.0% (0) | | 0.0% (0) | |
| Severe problems | 0.0% (0) | 0.0% (0) | | 0.0% (0) | |  | 0.0% (0) | 0.0% (0) | | 0.0% (0) | |
| Unable | 0.0% (0) | 0.0% (0) | | 1.0% (1) | |  | 0.0% (0) | 0.0% (0) | | 0.0% (0) | |
| **Pain/Discomfort** |  |  | |  | |  |  |  | |  | |
| No problems | 68.3% (69) | 81.0% (81) | | 76.8% (76) | |  | 61.2% (30) | 79.6% (39) | | 81.6% (40) | |
| Slight problems | 28.7% (29) | 15.0% (15) | | 17.2% (17) | |  | 32.7% (16) | 18.4% (9) | | 18.4% (9) | |
| Moderate problems | 2.0% (2) | 4.0% (4) | | 4.0% (4) | |  | 4.1% (2) | 2.0% (1) | | 0.0% (0) | |
| Severe problems | 1.0% (1) | 0.0% (0) | | 1.0% (1) | |  | 2.0% (1) | 0.0% (0) | | 0.0% (0) | |
| Extreme problems | 0.0% (0) | 0.0% (0) | | 1.0% (1) | |  | 0.0% (0) | 0.0% (0) | | 0.0% (0) | |
| **Anxiety/Depression** |  |  | |  | |  |  | |  | |  |
| No problems | 84.2% (85) | 93.0% (93) | | 87.9% (87) | |  | 79.6% (39) | 85.7% (42) | | 83.7% (41) | |
| Slight problems | 14.9% (15) | 6.0% (6) | | 11.1% (11) | |  | 16.3% (8) | 10.2% (5) | | 16.3% (9) | |
| Moderate problems | 1.0% (1) | 1.0% (1) | | 0.0% (0) | |  | 4.1% (2) | 4.1% (2) | | 0.0% (0) | |
| Severe problems | 0.0% (0) | 0.0% (0) | | 0.0% (0) | |  | 0.0% (0) | 0.0% (0) | | 0.0% (0) | |
| Extreme problems | 0.0% (0) | 0.0% (0) | | 1.0% (1) | |  | 0.0% (0) | 0.0% (0) | | 0.0% (0) | |
| EQ-5D Index Values (model from the Japan) | 0.90±0.11 (101) | 0.95±0.11 (100) | | 0.92±0.15 (99) | |  | 0.88±0.12  (49) | 0.93±0.11  (49) | | 0.92±0.12  (49) | |
| EQ Visual Analogue Scale | 77.4±14.7 (101) | 82.4±13.6 (100) | | 82.7±12.6 (99) | |  | 78.1±13.8 (49) | 81.0±13.4 (49) | | 83.2±10.4 (49) | |

Intent-to-treat subjects; Values are in % (n)

**Supplementary Table 2. Quality of Life (ISR Substudy)**

| **EQ-5D** | **Agent** | | |
| --- | --- | --- | --- |
|  | **Baseline**  **N=30 patients** | **6 months**  **N=29 patients** | **1 year**  **N=29 patients** |
| **Mobility** |  |  |  |
| No problems | 83.3% (25) | 86.2% (25) | 82.8% (24) |
| Slight problems | 3.3% (1) | 10.3% (3) | 6.9% (2) |
| Moderate problems | 6.7% (2) | 0.0% (0) | 3.4% (1) |
| Severe problems | 0.0% (0) | 0.0% (0) | 6.9% (2) |
| Unable | 6.7% (2) | 3.4% (1) | 0.0% (0) |
| **Self-Care** |  |  |  |
| No problems | 93.3% (28) | 86.2% (25) | 93.1% (27) |
| Slight problems | 3.3% (1) | 10.3% (3) | 3.4% (1) |
| Moderate problems | 0.0% (0) | 3.4% (1) | 3.4% (1) |
| Severe problems | 3.3% (1) | 0.0% (0) | 0.0% (0) |
| Unable | 0.0% (0) | 0.0% (0) | 0.0% (0) |
| **Usual Activities** |  |  |  |
| No problems | 76.7% (23) | 86.2% (25) | 86.2% (25) |
| Slight problems | 13.3% (4) | 6.9% (2) | 10.3% (3) |
| Moderate problems | 6.7% (2) | 3.4% (1) | 3.4% (1) |
| Severe problems | 0.0% (0) | 3.4% (1) | 0.0% (0) |
| Unable | 3.3% (1) | 0.0% (0) | 0.0% (0) |
| **Pain/Discomfort** |  |  |  |
| No problems | 70.0% (21) | 72.4% (21) | 69.0% (20) |
| Slight problems | 20.0% (6) | 13.8% (4) | 17.2% (5) |
| Moderate problems | 6.7% (2) | 3.4% (1) | 3.4% (1) |
| Severe problems | 3.3% (1) | 10.3% (3) | 10.3% (3) |
| Extreme problems | 0.0% (0) | 0.0% (0) | 0.0% (0) |
| **Anxiety/Depression** |  |  |  |
| No problems | 76.7% (23) | 82.8% (24) | 82.8% (24) |
| Slight problems | 16.7% (5) | 13.8% (4) | 13.8% (4) |
| Moderate problems | 6.7% (2) | 3.4% (1) | 0.0% (0) |
| Severe problems | 0.0% (0) | 0.0% (0) | 3.4% (1) |
| Extreme problems | 0.0% (0) | 0.0% (0) | 0.0% (0) |
| EQ-5D Index Values (model from the Japan) | 0.86±0.23 (30) | 0.88±0.20 (29) | 0.89±0.17 (29) |
| EQ Visual Analogue Scale | 75.8±21.1 (30) | 76.7±18.5 (29) | 82.3±14.5 (29) |

Intent-to-treat subjects; Values are in % (n)
